# Binding abilities of a chiral calix[4]resorcinarene: a polarimetric investigation on a complex case of study

**DOI:** 10.3762/bjoc.13.268

**Published:** 2017-12-15

**Authors:** Marco Russo, Paolo Lo Meo

**Affiliations:** 1 Dipartimento di Scienze e Tecnologie Biologiche, Chimiche e Farmaceutiche (STEBICEF), University of Palermo, V.le delle Scienze ed. 17, 90128 Palermo, Italy; 2 ATeNCenter, University of Palermo, V.le delle Scienze ed. 18, 90128 Palermo, Italy

**Keywords:** calix[4]resorcinarene, host–guest complexes, *p*-nitroanilines, polarimetry, supramolecular chemistry

## Abstract

Polarimetry was used to investigate the binding abilities of a chiral calix[4]resorcinarene derivative, bearing L-proline subunits, towards a set of suitably selected organic guests. The simultaneous formation of 1:1 and 2:1 host–guest inclusion complexes was observed in several cases, depending on both the charge status of the host and the structure of the guest. Thus, the use of the polarimetric method was thoroughly revisited, in order to keep into account the occurrence of multiple equilibria. Our data indicate that the stability of the host–guest complexes is affected by an interplay between Coulomb interactions, π–π interactions, desolvation effects and entropy-unfavorable conformational dynamic restraints. Polarimetry is confirmed as a very useful and versatile tool for the investigation of supramolecular interactions with chiral hosts, even in complex systems involving multiple equilibria.

## Introduction

During the last decades calix[*n*]arenes and calix[*n*]resorcinarenes (CAs) have emerged as versatile supramolecular host systems for various applications [1–5], spanning from sensors [6–7] to catalysis [8–9] and drug carriers [10–13]. Unlike the more popular cyclodextrins (CDs), CAs are exclusively obtained by chemical synthesis [14–18]. Therefore, they are particularly suitable for designing tailored systems with peculiar properties and abilities. This can be generally achieved by linking suitable donor groups to the aromatic scaffold. Among the virtually countless examples available in recent literature, L-proline-modified CAs constitute an interesting subject of study [19–32]. Proline-based systems in general have been proven excellent stereoselective organocatalysts [33–40]. In particular, CA derivatives bearing proline units (on both the upper and the lower rim) have been tested as catalysts for asymmetric aldol reactions in water [28–30,33]. Similar derivatives have also been studied as hydrogelators [22–23]. Moreover, water soluble chiral calix[4]resorcinarenes have been recently designed and used as chiral shift reagents for NMR applications [24–27].

The possibility to introduce chiral groups onto the CA scaffold is particularly intriguing from the viewpoint of the methodologies for investigating host–guest binding equilibria. In fact, simple polarimetry has been recently demonstrated to be an appealing and versatile tool for studying the host–guest interactions that imply cyclodextrins (CDs) [41–45], as well as for a reliable evaluation of the relevant binding constants. We were interested in verifying if the same technique could be suitably applied to other classes of chiral hosts. Thus, proline-modified calixarenes or calixresorcinarenes appeared ideal testing candidates. It is also worth noting that, because of the large variety of diversely modified CA derivatives existing, the binding abilities of these macrocycles have been subjected to less systematic and thorough studies [32,46–48] as compared to other classes of hosts such as CDs.

**Figure 1:**
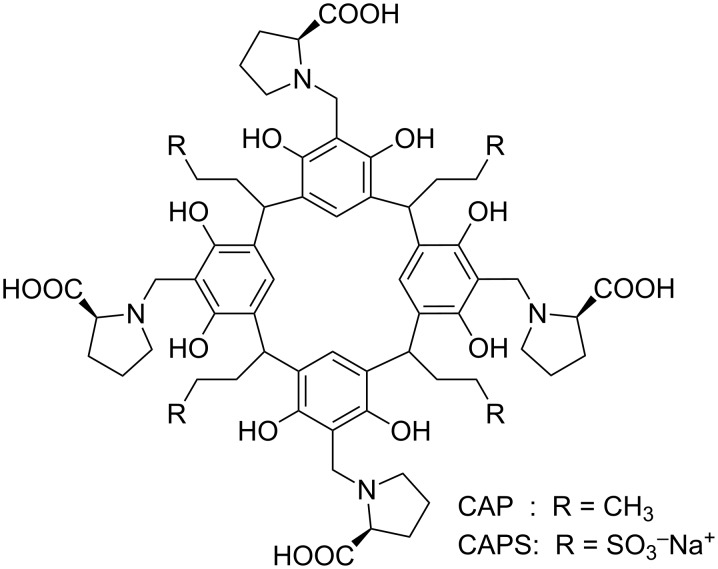
Structure of the L-proline-calix[4]resorcinarene derivatives CAP and CAPS.

**Figure 2:**
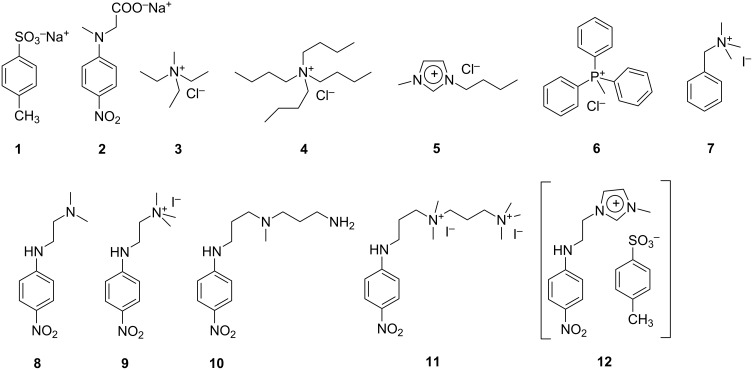
Structures of guests **1**–**12**.

With the aim at gaining a deeper understanding of the microscopic and thermodynamic aspects of the binding phaenomena involving CAs, as well as at verifying the possibility to extend the use of polarimetry as an investigation tool to these systems, in the present work we studied the binding abilities of an easily accessible L-proline-derivatized calix[4]resorcinarene, namely 2,8,14,20-tetrapropyl-4,6,10,12,16,18,22,24-octahydroxy[5,11,17,23-(L-prolin-1-yl)methyl]calix[4]resorcinarene (CAP, Figure 1) towards a set of variously structured organic guests **1**–**12** (Figure 2). The host CAP was designed in analogy with a sulfonated chiral calix[4]resorcinarene (CAPS, Figure 1) already known from the literature as NMR shift reagent able to perform chiral recognition [24–27]. Guests **1**–**12** were selected for their diverse structural features. We considered both neutral and ionic species, in particular aliphatic and aromatic cations of different size and hydrophobic character. Moreover, some *p*-nitroaniline derivatives were selected, because this class of molecules have been already proven as excellent probe guests to assess the microscopic interactions controlling the binding abilities of cyclodextrins [43–45,49–53].

## Results and Discussion

### Synthesis and solubility properties of CAP

**Figure 3:**
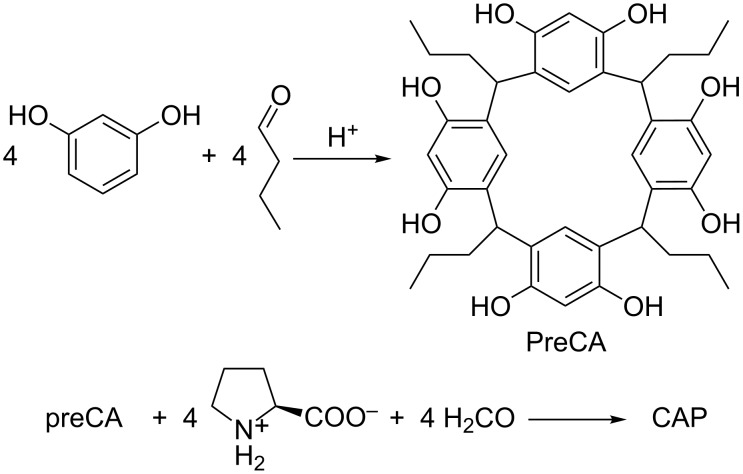
Synthesis of CAP.

As we mentioned previously, the synthesis of CAP was approached (see Experimental) in a similar way as the one reported for its sulfonate analogue CAPS [26], i.e., by subjecting the preformed (2,8,14,20-tetrapropyl)-(4,6,10,12,16,18,22,24-octahydroxy)calix[4]resorcinarene (preCA) [54] to a Mannich-type reaction with L-proline and formaldehyde (Figure 3).

The precursor preCA, in turn, was obtained by an acid-catalysed condensation between resorcinol and butyraldehyde. Of course, the main difference between the syntheses of CAP and CAPS is constituted by the choice of the starting aldehyde, namely simple butyraldehyde instead of a 3-sulfonatopropionaldehyde (which in turn must be generated in situ from commercial precursors). This derived from the need to rule out the occurrence of any possible interaction between cationic guests and the negatively charged pendant chains linked to the methylene bridges at the 2, 8, 14 and 20 positions of the macrocycle scaffold, specifically in order to address the interaction with the host cavity and, possibly, the pendant proline moieties. The structure of the final product was confirmed by NMR (see Supporting Information File 1 for details).

It is worth stressing here that, owing to the hydrophobic nature of the ancillary propyl groups, CAP is sparingly soluble in water under neutral conditions, whereas its solubility significantly increases as an increasing amount of a strong base is added. Noticeably, neutral CAP possesses 16 ionizable sites (four sites per prolinylarene subunit) and 12 acidic hydrogens, keeping into account both the proline moieties and the phenolic groups [32]. One can reasonably expect that the proline subunits at the macrocycle’s upper rim are present in their zwitterionic form. On grounds of the p*K*_a_ values reported in the literature [55] for free proline (1.95, 10.64) and resorcinol (9.32, 11.1), phenolic groups appear the most acidic, although deprotonation of the proline units cannot be excluded a priori. It is worth mentioning here that for a L-proline-calixresorcinarene derivative very similar to CAP (with methyl groups in place of the *n*-propyl groups at the methylene bridges), an average p*K*_a_ value as large as 6.3 ± 1 per arene subunit has been estimated from titration curves, under the hypothesis that the four subunits behave equivalently [32]. In the latter case, the deprotonation of phenol groups (which appear more acidic than expected because of intramolecular hydrogen bonding) was supported by NMR evidences.

Owing to solubility issues, we addressed our interest in evaluating the binding abilities of the anionic forms of CAP, namely the mono-, di-, tri- and tetra-anion, which could be obtained, in principle, by simply adding the proper stoichiometric amount of a strong base (i.e., one, two, three or four equivalents of NaOH) to a suspension of the host. However, because of the chemical equivalence of the four prolinylarene subunits, from an analytical viewpoint the addition of a given amount of base cannot result in the exclusive formation of the desired anionic form alone, but rather in a mixture of differently charged anions at equilibrium. Of course, the average charge of the anionic species formed equals the number of base equivalents added. Moreover, it can be algebraically shown that the prevailing anion is actually the ideal one that corresponds to the number of base equivalents added. Thus, it is reasonable, as a first approximation, to consider that a system formed by mixing CAP with a given amount of base equates in its properties the corresponding ideal anion. Hereinafter, we will refer to the systems obtained by mixing one, two, three or four equivalents of base to CAP as CAP^−1^, CAP^−2^, CAP^−3^, and CAP^−4^, respectively.

### Polarimetry: methodological issues

Before examining the results of our polarimetric investigations on CAP and its complexes, few methodological clarifications must be provided (extensive discussion can be found in Supporting Information File 1). According to literature [41–42], the use of polarimetry to study binding equilibria requires the preparation of a set of samples, by mixing a fixed amount (*V*_0_) of a solution of the host with increasing micro-amounts (*v*_i_) of a concentrated solution of the guest (method A, see Experimental). Then, under the hypothesis that only 1:1 complexes are formed it can be algebraically shown that the optical activities 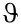
_i_ of the samples must vary according to the relationship in Equation 1.

[1]
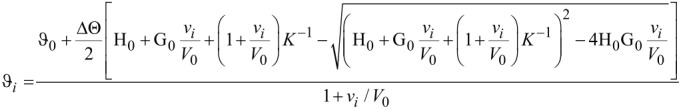


In Equation 1 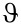
_0_ is the optical rotation of the pure host solution, ΔΘ is the differential molar optical rotation (i.e., the difference between the molar optical rotations of the complex and the free host, respectively), H_0_ and G_0_ are the concentrations of the host and guest mother solutions, respectively, *K* is the required binding constant. As we will discuss in detail later, complexation of substrates **1**–**12** with CAP leads in several cases to the formation of 2:1 complexes, either alone or together with the corresponding 1:1 complexes (as accounted for by the analysis of the relevant Job plots). In these cases, Equation 1 cannot be used for data regression analysis and the entire problem must be completely readdressed.

When both complexes are simultaneously formed, according to the equilibria:


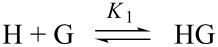


and


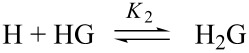


the optical activity of a generic *i*-th sample is given by the relationship:

[2]
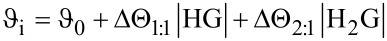


In Equation 2 ΔΘ_1:1_ and ΔΘ_2:1_ are the differential molar optical rotations of the two complexes (i.e., ΔΘ_1:1_ = Θ_HG_ − Θ_H_ and ΔΘ_2:1_ = Θ_H2G_ − 2Θ_H_; Θ_H_, Θ_HG_, and Θ_H2G_ are the molar optical rotations of the free host, the 1:1 and the 2:1 complexes, respectively), |HG| and |H_2_G| are the concentrations of the complexes at equilibrium. Hence, by applying the required mass balances and equilibrium conditions, one finally obtains:

[3]
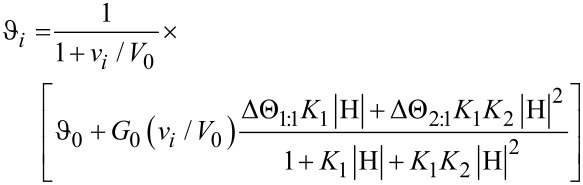


In Equation 3 *K*_1_ and *K*_2_ are the required binding constants, and |H| is the concentration of the free host at equilibrium, which in turn can be calculated by solving the equation:

[4]
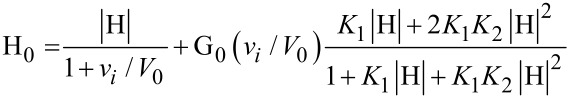


Unfortunately, Equation 4 reduces to a cubic form; thus, Equation 3 cannot be solved analytically and is unsuitable for data regression analysis. The problem can be smartly worked out by means of an iterative approach. In fact, Equation 4 can be transformed as:

[5]
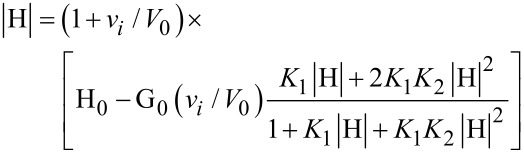


Therefore, having fixed H_0_ and G_0_ values and two first-approximation test values for *K*_1_ and *K*_2_, Equation 5 can be iteratively solved to obtain a first approximation set of |H| values for the samples. Then, by reporting 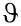
_i_ vs |H|, Equation 3 can be used as fitting equation to obtain two second-approximation values for *K*_1_ and *K*_2_. The latter ones are reinserted into the Equation 5 to re-calculate |H| values, and the entire procedure is iterated up to convergence. Of course, from *K*_1_ and *K*_2_ values the relevant cumulative stability constant β_2_ can be easily calculated as: β_2_ = *K*_1_*K*_2_. Moreover, if only 2:1 complexes are formed (as accounted for by the Job plot), i.e., whenever the stability of the 2:1 complex is so high that the 1:1 complex is never formed in appreciable amount under the experimental conditions used, *K*_1_ cannot be evaluated and Equation 3 and Equation 5 can be easily simplified accordingly (see Supporting Information File 1).

Finally, for the sake of completeness, it must be mentioned here that samples can be alternatively prepared by mixing the host solution with increasing weighed amounts of the solid guest (method B, see Experimental). Even in the latter case, of course, Equations 3–5 can be suitably adapted (see Supporting Information File 1 for details).

### Polarimetric properties of CAP

**Figure 4:**
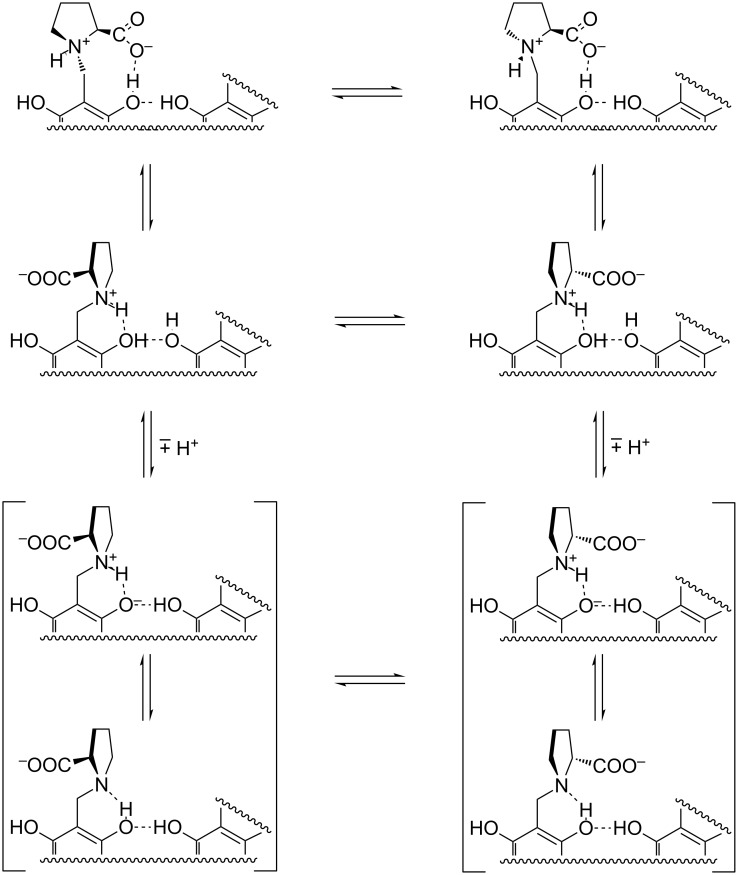
Structural models for the conformational rearrangements of CAP.

As a preliminary work, we evaluated the polarimetric response of the anionic forms of CAP (in the sense discussed above). Noticeably, the addition of NaOH to the suspension of pristine CAP always resulted in the formation of clear solutions under the concentration conditions used. We found that CAP^−1^ is dextrorotatory, with a molar optical rotation Θ_1_ as large as +6.5 ± 0.1 deg dm^−1^ M^−1^; by contrast, CAP^−2^, CAP^−3^ and CAP^−4^ resulted laevorotatory, with molar optical rotation values as large as Θ_2_ = −19.3 ± 0.4 deg dm^−1^ M^−1^, Θ_3_ = −20.1 ± 0.3 deg dm^−1^ M^−1^, Θ_4_ = −21.5 ± 0.4 deg dm^−1^ M^−1^, respectively. These results appear quite interesting when compared with the value of the molar optical rotation of *N*-benzyl-L-proline, which can be deduced from literature data [56], namely −19.9 deg dm^−1^ M^−1^. If the optical activity of the macrocycle would merely depend on the presence of the amino acid moieties, then a molar optical rotation as large as ca. −80 deg dm^−1^ M^−1^ should be expected. By analogy with what observed for polysaccharides [41–42], differences with the observed values might be in principle ascribed to either electronic effects, or conformational rearrangements of the overall macrocycle structure. However, the fact that Θ_2_, Θ_3_ and Θ_4_ values are similar indicates that extensive deprotonation of the macrocycle has a minor outcome; therefore, a significant contribution from electronic effects may be ruled out. On the other hand, large conformational rearrangements deriving from progressive deprotonation, and the consequent presence of an increasing negative charge, are reasonable. It is worth recalling here that the cone conformation of the resorcinarene scaffold is stabilized by the possible formation of a hydrogen-bond network between pairs of phenol groups on adjacent arene units [32]. Trivial molecular models (Figure 4) easily show that both the nitrogen atom and the carboxylate group of the proline units can participate in this network by donating or accepting hydrogen bonds.

**Figure 5:**
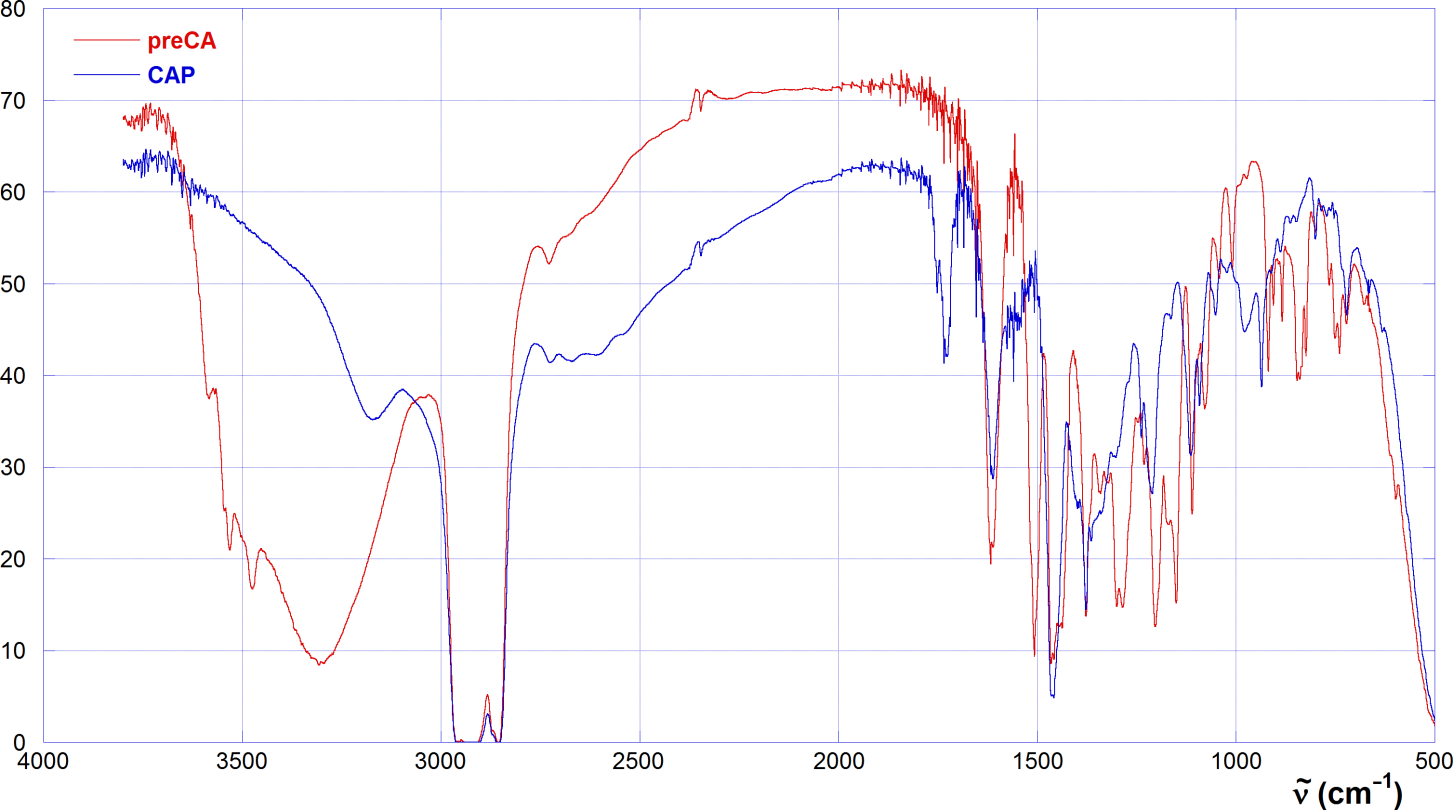
FTIR spectra of preCA (red) and CAP (blue).

This hypothesis is supported by analysis of FTIR spectra (Figure 5). In fact, preCA shows the typical intense and large band for the O–H stretching centred at 3304 cm^−1^, accompanied by two tiny additional signals at 3534 and 3476 cm^−1^. After attachment of the proline units, the spectrum of CAP shows a significant decrease in intensity and a bathochromic shift to 3169 cm^−1^ of the O–H band; moreover, a carbonyl band of fair intensity appears at 1727 cm^−1^, similar to the one expected for an undissociated carboxylic group. In turn, extensive hydrogen bonding affects the flexibility of the macrocycle scaffold, as well as the possible double free rotation of the arene–CH_2_–proline single bond–single bond system. Hydrogen bonding is likely enforced by deprotonation of a phenol group, due to enhanced Coulomb interaction with ammonium groups. Moreover, protonation of the nitrogen atom makes it a further chiral centre, which contributes to the overall optical activity of the system. Then, both the absolute configuration assumed by the protonated N atom and the conformation of the arene–CH_2_–proline double free-rotating system, determine in turn the relative position of the negatively charged and bulky carboxylate group with respect to the macrocycle cavity (i.e., inwards or outwards). This provides a further contribution to the overall dissymmetry of the host. Everything considered, polarimetric results indicate that CAP undergoes some major structural rearrangement specifically on passing from the mono- to the dianion form, probably due to the occurrence of a severe reduction of the conformational freedom for the arene–CH_2_–proline system. Subsequent proton loss simply results in further stabilization of the overall conformation assumed by the dianionic form.

As a final remark, it is worth stressing that, irrespective of the amount of base added, the optical activity of a solution of CAP is lost within a couple of days, even if stored at low temperature (4 °C). Therefore, the proline subunits easily undergo racemization under alkaline conditions.

### Binding properties of CAP

Based on the previous results, we preliminarily tested the interaction of guests **1**–**12** with CAP^−2^. We observed that anions **1** and **2** and aliphatic cations **3** and **4** do not appreciably interact with the host. Lack of binding with the anions can be easily attributed to the occurrence of unfavourable Coulomb repulsion. On the other hand, the fact that even cations **3** and **4** do not show appreciable affinity for the host clearly outlines an important role assumed by π–π interactions. In fact, the small aromatic imidazolium cation **5** is appreciably included into CAP^−2^, although with a relatively small binding constant (*K* = 250 ± 20 M^−1^; ΔΘ = −14.6 ± 0.4 deg dm^−1^ M^−1^). It is interesting to notice that significant affinity towards CAP^−2^ is also shown by neutral nitroaniline derivatives **8** and **10**, providing further confirmation that π–π interactions play an important role. Nevertheless, it is also worth noting here that the importance of electrostatic effects has been already outlined by Schneider and Schneider [32], who examined the behaviour of diverse calix[4]resorcinarenes, including a proline derivative very similar to CAP. In particular, it was observed that the relevant octo-anion shows remarkable affinity towards aliphatic ammonium cations (as well as the tetra-anion of the title ligand bearing no proline groups), whereas the binding properties of the tetra-anion are only fair. Then, on the grounds of these preliminary data, guests **6**–**12** were selected for a more detailed study considering also the other anionic forms of the host. The complete results are collected in Table 1.

**Table 1:** Binding constants for CAP anions with guests **6**–**12**.

	CAP^−1^		CAP^−2^		CAP^−3^		CAP^−4^	

guest	*K*_1_ (10^3^ M^−1^)	ΔΘ_1:1_ (deg dm^−1^ M^−1^)	*K*_1_ (10^3^ M^−1^)	ΔΘ_1:1_ (deg dm^−1^ M^−1^)	*K*_1_ (10^3^ M^−1^)	ΔΘ_1:1_ (deg dm^−1^ M^−1^)	*K*_1_ (10^3^ M^−1^)	ΔΘ_1:1_ (deg dm^−1^ M^−1^)

**6**	13 ± 4	−6.6 ± 1.0	10.5 ± 0.4	−21.9 ± 0.9	3.9 ± 0.4	−25.8 ± 0.4	1.74 ± 0.14	−40.0 ± 0.6
**7**	–	–	1.35 ± 0.14	−16.8 ± 0.3	0.82 ± 0.08	−42.3 ± 0.8	0.58 ± 0.04	−67 ± 1
**8**	3.5 ± 0.4	−97 ± 9	–	–	–	–	0.51 ± 0.13	−6.5 ± 0.6
**9**	(<0.2)	(>0)	–	–	4.5 ± 0.3	−59 ± 3	2.1 ± 0.3	−91 ± 2
**10**	5 ± 2	−82 ± 6	5.8 ± 0.9	−67 ± 6	15.2 ± 1.1	−28 ± 2	0.19 ± 0.02	−16.9 ± 0.8
**11**	(<0.2)	(>0)	–	–	–	–	13.4 ± 0.5	−124 ± 8
**12**	(<0.2)	(>0)	42 ± 6	−4.9 ± 0.8	2.5 ± 0.8	−39 ± 2	1.05 ± 0.15	−61 ± 2

	β_2_ (10^6^ M^−2^)	ΔΘ_2:1_ (deg dm^−1^ M^−1^)	β_2_ (10^6^ M^−2^)	ΔΘ_2:1_ (deg dm^−1^ M^−1^)	β_2_ (10^6^ M^−2^)	ΔΘ_2:1_ (deg dm^−1^ M^−1^)	β_2_ (10^6^ M^−2^)	ΔΘ_2:1_ (deg dm^−1^ M^−1^)

**6**	13 ± 3^a^	34 ± 3	19 ± 2^b^	−33.2 ± 0.9	–	–	–	–
**7**	–	–	–	–	–	–	–	–
**8**	(<1)	(>0)	1.05 ± 0.13	−63 ± 3	4.8 ± 0.3	−90 ± 3	–	–
**9**	–	–	2.85 ± 0.15	−48.7 ± 1.0	1.36 ± 0.19^c^	−100 ± 10	–	–
**10**	–	–	(<1)	(<0)	1.3 ± 0.4^d^	−72 ± 3	–	–
**11**	–	–	1.25 ± 0.05	−61 ± 4	1.52 ± 0.14	−200 ± 4	17.1 ± 1.8^e^	−200 ± 3
**12**	–	–	80 ± 20^f^	−16.7 ± 0.9	–	–	–	–

^a^*K*_2_ = (1.0 ± 0.1)·10^3^ M^−1^; ^b^*K*_2_ = (1.8 ± 0.2)·10^3^ M^−1^; ^c^*K*_2_ = (0.3 ± 0.1)·10^3^ M^−1^; ^d^*K*_2_ = (0.9 ± 0.2)·10^3^ M^−1^; ^e^*K*_2_ = (1.3 ± 0.1)·10^3^ M^−1^; ^f^*K*_2_ = (1.9 ± 0.3)·10^3^ M^−1^.

As a first observation, we can notice that the binding abilities of CAP^−1^ are modest. Significant inclusion is shown only by the large triphenylphosphonium cation **6**, and by the neutral guests **8** and **10**. In the former two cases, even the presence of 2:1 complexes can be detected. Polarimetric data for cations **9**, **11** and **12** reveal a weak interaction, but do not allow a reliable estimation of the binding constant, whereas the benzylammonium cation **7** does not interact at all. By contrast, CAP^−2^ and CAP^−3^ include very well all the guests, forming in several cases the relevant 2:1 complexes. More in detail, the exclusive formation of the 1:1 complex is found only in four cases out of fourteen, whereas in five cases both complexes are formed, and in five cases the 2:1 complex is exclusively found. Finally, CAP^−4^ forms 1:1 complexes with all the guests (only **11** forms both complexes). Therefore, we can conclude that the observed stoichiometry of the aggregates is not affected by the charge status of the host in a simple way, although data clearly suggest that the tendency to form 2:1 complexes decreases on increasing the charge of the host. The latter observation can be easily justified assuming the occurrence of a head-to-head arrangement for the 2:1 complex, which is strongly destabilized for CAP^−4^ due to the occurrence of rim-to-rim electrostatic repulsion. Size, shape and charge status of the guest, of course, play a paramount role in determining both the stoichiometry and the stability of the aggregates. For instance, the small benzylammonium cation **7** forms 1:1 complexes only. In this case, *K*_1_ values decrease on increasing the average negative charge of the host (whereas CAP^−1^ does not include it, as we already mentioned). If Coulomb interactions were the main driving force for the inclusion process, then a regular increase of *K*_1_ values would have been observed. On the other hand, desolvation of the host is expected to become more and more difficult on increasing its charge status. Moreover, it is interesting to notice that the relevant ΔΘ_1:1_ values become more and more negative on increasing host charge. According to literature [41–45], this indicates the occurrence of severer and severer dynamic-conformational changes upon complex formation. Therefore, we can conclude that the overall bell-shaped trend for *K*_1_ values on passing from CAP^−1^ to CAP^−4^ is the outcome of a fine interplay between favourable electrostatic factors and unfavourable desolvation and entropic effects, with CAP^−2^ benefitting from the best compromise among them. Noticeably, as long as 1:1 complexes are concerned, close inspection of data reported in Table 1 shows that the same increasing trend for ΔΘ_1:1_ absolute values also occurs for mono-cations **6**, **9** and **12** (the dication **11** cannot be compared, because it forms only the 2:1 complexes with CAP^−2^ and CAP^−3^). However, the relevant trends for *K*_1_ values are slightly different. Similarly to guest **7**, the imidazolium cation **12** shows a bell-shaped trend with its maximum for CAP^−2^ (the *p*-toluensulfonate counteranion does not interact with the host), whereas for the ammonium cation **9** the largest *K*_1_ is found with CAP^−3^ (in the latter case, however, the datum for the CAP^−2^ is lacking, because only the 2:1 complex is observed). By contrast, the complexes of the bulky triarylphosphonium derivative **6** monotonically decrease in stability on increasing the average host charge, likely due to its high hydrophobic character. Among the neutral guests, only **10** presents the complete set of the 1:1 complexes; even in this case, *K*_1_ values show a bell-shaped trend, with a maximum value for CAP^−3^. It is interesting to notice that ΔΘ_1:1_ values for neutral guests show a decreasing trend in their absolute values on increasing the charge of the host, in striking contrast with the behaviour observed for cationic guests.

On passing to analyse the results relevant to the 2:1 complexes, we must preliminary notice that with no guest it is possible to find the complete set of data with all the four differently charged forms of the host. At the best, the dication **11** lacks only the 2:1 complex with CAP^−1^. Both β_2_ and ΔΘ_2:1_ absolute values for this guest increase on increasing the negative charge of the host. For the bulky cation **6** the 2:1 complexes can be found with CAP^−1^ and CAP^−2^, whereas nitroaniline derivatives **8** and **9** form stable 2:1 complexes with CAP^−2^ and CAP^−3^. Clear trends for the stability of the complexes cannot be envisaged. In fact, β_2_ increases as the charge of the host increases for cations **6** and **9**, whereas the opposite is observed with the neutral **8**, indicating a clear contribution from Coulomb interactions. This is confirmed by the fact that the dication **11** is the only guest able to afford the 2:1 complex with CAP^−4^, due clearly to the fact that its double charge can effectively counterbalance the Coulomb repulsion between the two host units. Noticeably, in all these cases ΔΘ_2:1_ values become more negative on increasing the charge of the host. This is particularly apparent for **6**, the ΔΘ_2:1_ values of which pass from positive to negative on passing from CAP^−1^ to CAP^−2^. Finally, the neutral guest **10** forms a stable 2:1 complex only with CAP^−3^, whereas the imidazolium derivative **12** forms a 2:1 complex with CAP^−2^. The whole of these results suggests that the stability of a possible 2:1 complex requires once again a compromise between several factors, and that the optimum conditions largely vary depending on the structure of the guest. It is worth noting that in six cases out of eleven the values of the binding constants *K*_2_ (see footnote of Table 1) are numerically smaller than the relevant *K*_1_, indicating that the 2:1 complex is intrinsically less stable than the 1:1 one. On the other hand, in the five cases where only the 2:1 complex is detected, this implies a much higher stability as compared to the 1:1 complex. A simple numeric analysis (see Supporting Information File 1 for details) suggests that in these cases *K*_2_ values should be larger than 8·10^3^ M^−1^ (and consequently *K*_1_ lower than 1·10^3^ M^−1^).

Owing to the diverse behaviours observed, a comparison between the different guests is not straightforward, and a full rationalization of the outcome of their structural features on the binding equilibrium is not simple. Nevertheless, the data allow some further interesting observations. In particular, the fact that the largest *K*_1_ values are found with the bulkiest guests **6**, **11** and **12** confirms that hydrophobic effects and π–π interactions are as much important as Coulomb interactions in determining the stability of the complex. On the other hand, neutral guests **8** and **10** are not comparable in behaviour with the relevant cations **9** and **11**, respectively. Moreover, as long as ΔΘ_1:1_ values for CAP^−4^ are concerned (the only case for which the complete data set with all guests is available), no strict relationship with the guest bulkiness can be envisaged, even if cationic and neutral guests are considered separately. This suggests that the conformational and dynamic restrictions consequent to inclusion may be due to the occurrence of specific host–guest interactions rather than to a mere steric effect.

**Figure 6:**
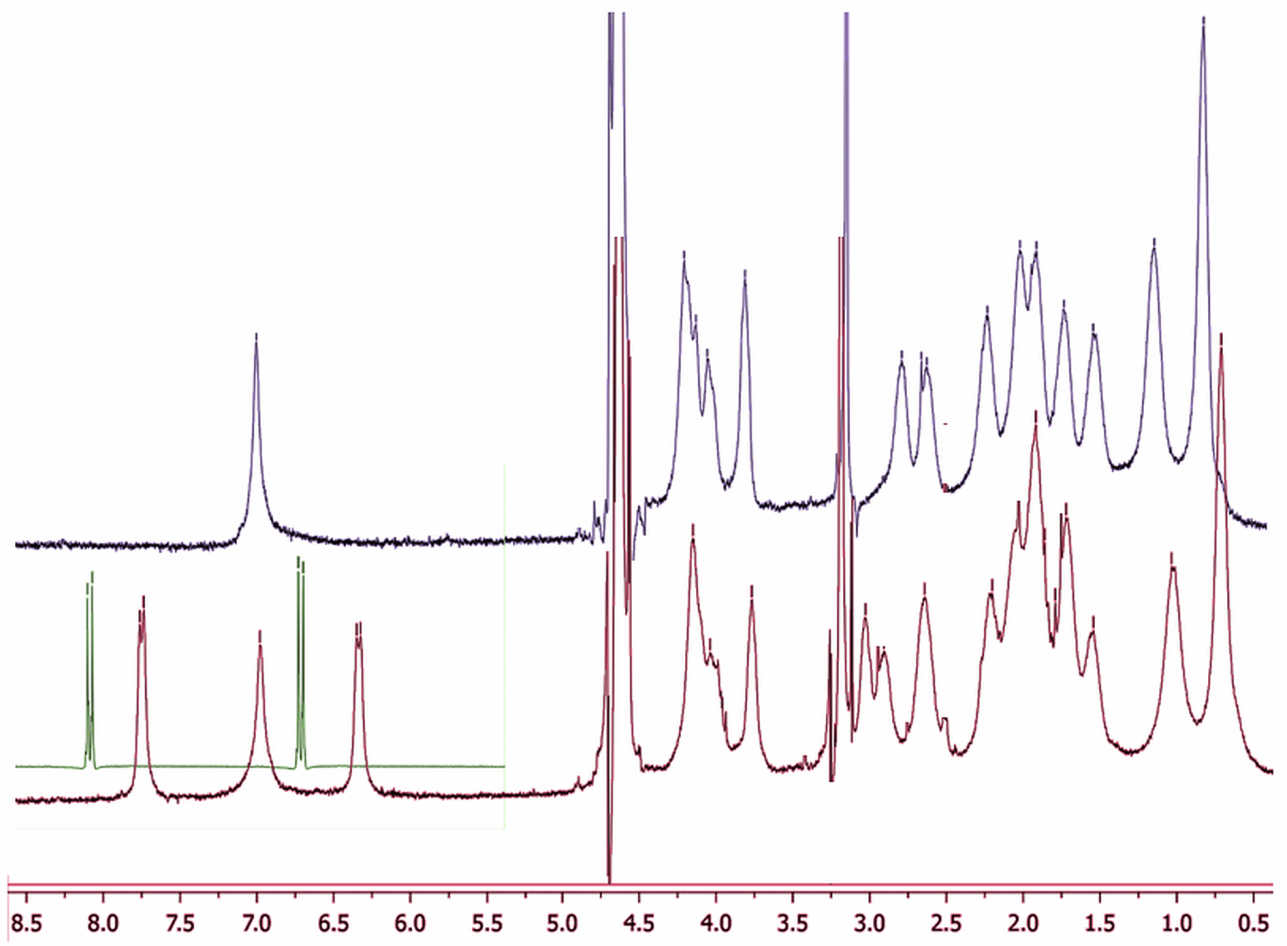
^1^H NMR spectra (D_2_O) spectra of CAP^−1^ (blue), **8** (green, aromatic region only) and their 1:1 complex (purple).

In order to clarify the latter point, we investigated the possible structure of the complexes with *p*-nitroaniline derivatives by means of NMR techniques. In particular, the ^1^H spectrum of the **8**·CAP^−1^ 1:1 complex (Figure 6) shows a large upfield shift (and loss of resolution) of the signals relevant to the aromatic H atoms of the guest (the signals relevant to the aliphatic moiety are deeply buried under those of the host, and cannot be identified). This indicates that the *p*-nitrophenyl group is allocated in the deshielding region provided by the aryl subunits of the host. Therefore, we can conclude that the aromatic moiety of the guest is specifically included into the cavity, in a quite similar way as the one occurring for the complexes of the same guests with cyclodextrins [49]. Regarding the signals relevant to CAP, the positions of the aromatic H at ca. 7.0 ppm and the proline H(3–5) atoms in the region between 1.60 and 2.70 ppm remain almost unchanged. On the other hand, a significant downfield shift and splitting is shown by the proline-H(2) signal (from 2.79 to 2.91–3.03 ppm), whereas a fair upfield shift is found for one of the *N*-methylene H atoms (from 3.81 to 3.77 ppm), which account for the possible conformational restraints on the proline-decorated rim occurring upon complex formation.

**Figure 7:**
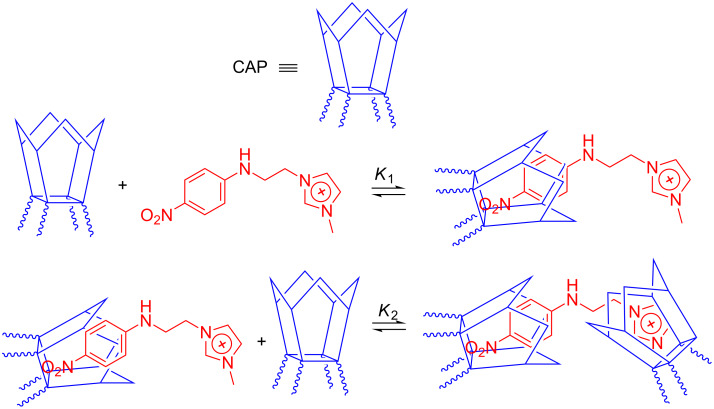
Possible depiction of the 1:1 and 1:2 complexes of **12**.

Interestingly, a fair upfield shift is observed also for the signals at 0.82 and 1.14 ppm (passing to 0.71 and 1.03 ppm, respectively) relevant to the propyl pendant groups linked at the 2, 8, 14 and 20 positions of the calixarene scaffold. Assuming for CAP the occurrence of an “*all-endo*” stereochemistry (i.e., according to the terminology introduced by Högberg [57–58], the thermodynamically most stable “*cis-cis-cis*” structure, see Supporting Information File 1), trivial molecular models show that in the free host they can easily access the deshielding region provided by the macrocycle cavity. Consequently, the inclusion of the guest forces them in a conformation that is more exposed to the solvent bulk. Finally, taking back to the guest, the inclusion of its *p*-nitrophenyl group into the cavity implies that the aliphatic moiety protrudes out of the proline-decorated host rim, interacting with it and affecting its conformational dynamism. Of course, the protruding moiety can subsequently interact with a second host unit to form the 2:1 complex. It is worth stressing that the most stable 2:1 complexes are once again formed by guests **6** and **12**, which possess more than one aromatic subunit. The case of the imidazolium derivative **12** is intriguing, because in principle its 1:1 complex might involve the inclusion of either aromatic ring. However, the preferential inclusion of the *p*-nitrophenyl group may be reasonably presumed on the grounds of the fact that the complex formed by the simple imidazolium cation **5** with CAP^−2^ is by far less stable than the ones formed by simple *p*-nitrophenyl derivatives **8** and **9** (a possible depiction of the complexes formed by **12** is shown in Figure 7).

## Conclusion

By means of a smart use of the polarimetric method, the binding equilibria between a chiral L-proline-derivatized calix[4]resorcinarene and a set of selected organic guests were studied. Our investigation was complicated by the fact that, depending on the structure of the guest, the simultaneous presence of different complexes, i.e., having a 1:1 and a 2:1 stoichiometry, may occur. In both cases, indeed, the inclusion seems controlled by a fine compromise between different factors. Two main driving forces of the inclusion process can be identified, namely: i) π–π interaction between the aromatic moiety of the guest and the host cavity, as accounted for by the scarce or negligible affinity observed of purely aliphatic cations; ii) non-specific electrostatic interactions, as accounted for by the lack of inclusion of anions. Furthermore, at least in the case of the *p*-nitroaniline derivatives, the aliphatic moiety of the guest interacts with the prolinylmethyl groups at the host rim, affecting their conformational dynamism, and consequently determining the actual polarimetric response. Along with non-specific steric, desolvation and electrostatic factors, also specific interactions may take place, the mutual interplay of which is hardly predictable, giving rise to the observed non-monotonic trends. The mutual balance between all these factors critically depends on the structure of the guest, in terms of its steric bulk, number of aromatic moieties and electric charge. This situation somehow resembles the one occurring for CDs; in fact, it has been largely demonstrated that the entire macrocylcle structure of the CD host is flexible enough to apt itself upon the guest molecule and optimize microscopic interactions [59]. In the case of CAP, polarimetric evidences rather suggest the idea that the main arene scaffold is fairly rigid, whereas actual structural rearrangements mainly involve the prolinylmethyl units at the rim. Nevertheless, polarimetric results positively indicate that the conformational dynamic changes of the host structure are not simply due to mere steric effects.

The results presented in this work provide a contribution to a deeper understanding of the microscopic interactions occurring in host–guest complex formation processes involving calixarenes in general. This can be particularly useful, even because CAP and structurally related ligands might find various interesting applications, due to their amphiphilic character, chirality and coordination ability towards metal cations [31–32], for instance as chiral selectors or as catalysts in micro-heterogeneous or organized systems (micelles, Langmuir–Blodgett films, ionic liquids etc.). Finally, our study shows how the use of polarimetry, which has already been shown a powerful tool for the systematic study of the binding abilities of CDs, can be profitably extended to other classes of chiral hosts, even in those cases in which multiple equilibria occur, provided that the relevant mathematical problems are suitably addressed.

## Experimental

All the reagents and materials needed were used as purchased (Aldrich, Fluka), without further purification. Non-commercial guests **2** and **8**–**12** were prepared according to literature [49,52–53]. The synthesis and characterization of preCA and CAP is reported in Supporting Information File 1. FTIR spectra were recorded with an AGILENT Cary 630 FTIR instrument; NMR spectra were acquired on a Brucker AS Series 300 MHz spectrometer, and polarimetric measurements were performed with a JASCO P-1010 polarimeter.

Stock solutions (2.5 mM) of the host at the required charge status were prepared by suspending 87.3 mg of CAP (75 μmol) in ca. 20 mL of double-distilled water. Then, the proper amount of a standard 1 M NaOH solution was added (i.e., 0.75 mL, 1.50 mL, 2.25 mL or 3.00 mL for CAP^−1^, CAP^−2^, CAP^−3^ and CAP^−4^, respectively). The suspension quickly turned into a clear solution, the volume of which was finally adjusted to 30 mL. The solution was used within few hours to avoid racemization of the L-proline subunits. Then, for each guest, a set of samples were prepared according to either of the following methods. Method A: to 3 mL of host stock solution, increasing amounts (up to 150 μL) of a 0.2 M solution of the guest in methanol were added. Then, the measured optical activity of the samples was subjected to regression analysis according to Equations 3–5. Method B: increasing weighed amounts (up to 8 mg) of the solid guest were dissolved with 3 mL of the host stock solution. The equations for data regression analysis were suitably adapted (see Supporting Information File 1 for details).

In order to record the ^1^H NMR spectrum of the **8**·CAP^−1^ complex, CAP (11.6 mg, 10 μmol) was dissolved in methanol (10 mL). Then 100 μL of NaOH (0.1 M) and **8** (2.1 mg, 10 μmol) were added. The resulting solution was stirred for 15 min, and then distilled in vacuo (Rotavapor). The residue was finally dissolved in D_2_O (1 mL).

## Supporting Information

File 1 Mathematical details on the derivation of the equations used for polarimetric data analysis, and the synthesis and charcaterization of preCA, CAP and the **8**·CAP^−1^ complex.
