# Supplementary material for: Binding abilities of a chiral calix[4]resorcinarene: a polarimetric investigation on a complex case of study
Source: Beilstein J Org Chem. 2017 Dec 15;13:2698–709. doi: 10.3762/bjoc.13.268 (PMC5753176; doi:10.3762/bjoc.13.268)
Supplement: File 1 — Mathematical details on the derivation of the equations used for polarimetric data analysis, and the synthesis and charcaterization of preCA, CAP and the 8·CAP−1 complex. [file Beilstein_J_Org_Chem-13-2698-s001.pdf]

Supporting Information

for

Binding abilities of a chiral calix[4]resorcinarene: a polarimetric investigation on a complex case of study

Marco Russo[1] and Paolo Lo Meo*[1,2]

Address: [1]Dipartimento di Scienze e Tecnologie Biologiche, Chimiche e Farmaceutiche (STEBICEF), University of Palermo, V.le delle Scienze ed. 17, 90128 Palermo, Italy and [2]ATeNCenter, University of Palermo, V.le delle Scienze ed. 18, 90128 Palermo, Italy

Email: Paolo Lo Meo - paolo.lomeo@unipa.it

* Corresponding author

Mathematical details on the derivation of the equations used for polarimetric data analysis, and the synthesis and charcaterization of preCA, CAP and the 8·CAP^{-1} complex

Derivation of the equations used for the application of the polarimetric method

As reported in literature (see [S1,S2]), Equation 1 is the exact solution of the three-variables problem (in |H|, |G| and |HG|) relevant to the formation of the 1:1 complex. This solution is obtained introducing no simplification in the equation system constituted by the stoichiometric conditions on H_0 and G_0 and the mass action condition: $|HG| = K|H||G|$. Of course, it can be used when the samples set is prepared by mixing the components in solution (method A, see Experimental). On the other hand, when samples are prepared by adding the guest as a solid to the solution of the host (method B), under the hypothesis that volume variations are negligible (i.e., that the partial molar volume of the guest is negligible), Equation 1 can be suitably simplified as follows:

$$\vartheta_i = \vartheta_0 + \frac{\Delta\Theta}{2}\left[H_0 + \frac{g_i}{V_0} + K^{-1} - \sqrt{\left(H_0 + \frac{g_i}{V_0} + K^{-1}\right)^2 - 4H_0\frac{g_i}{V_0}}\right] \quad \text{(S1)}$$

where g_i is the amount of guest in moles. When 1:1 and 2:1 complexes are simultaneously formed, we have to deal with a four-variables problem (in |H|, |G|, |HG| and $|H_2G|$). Therefore, the following equation system must be solved, constituted by the stoichiometric and mass action conditions:

$H_{0,i} = |H| + |HG| + 2|H_2G|$

$G_{0,i} = |G| + |HG| + |H_2G|$

$|HG| = K_1|H||G|$

$|H_2G| = \beta_2|H|^2|G| = K_1K_2|H|^2|G|$

Noticeably, if samples are prepared according to method A, then for the generic *i*-th sample we have $H_{0,i} = H_0/(1 + v_i/V_0)$ and $G_{0,i} = G_0 (v_i/V_0)/(1 + v_i/V_0)$; alternatively, if samples are prepared according to Method B, then $H_{0,i} = H_0$ and $G_{0,i} = g_i/V_0$.

The second equation of the system easily transforms as:

$G_{0,i} = |G| + |HG| + |H_2G| = |G| + K_1 |H||G| + K_1K_2 |H|^2|G| = |G| (1 + K_1|H| + K_1K_2|H|^2)$

Then:

$|G| = G_{0,i}/(1 + K_1|H| + K_1K_2|H|^2)$ (S2a)

$|HG|= G_{0,i} K_1|H|/(1 + K_1|H| + K_1K_2|H|^2)$ (S2b)

$|H_2G| = G_{0,i} K_1K_2|H|^2/(1 + K_1|H| + K_1K_2|H|^2)$ (S2c)

Consequently, we easily obtain:

$H_{0,i} = |H| + |HG| + 2|H_2G| =$

$= |H| + G_{0,i}\, K_1|H|/(1 + K_1|H| + K_1K_2|H|^2) + 2G_{0,i}\, K_1K_2|H|^2/(1 + K_1|H| + K_1K_2|H|^2) =$

$= |H| + G_{0,i}(K_1|H| + 2\, K_1K_2|H|^2)/(1 + K_1|H| + K_1K_2|H|^2)$ (S3)

If samples are prepared according to method A, Equation S3 immediately turns into Equation 4 after proper substitution of $H_{0,i}$ and $G_{0,i}$. On the other hand, if samples are prepared according to method B, after proper substitution of $H_{0,i}$ and $G_{0,I}$ one obtains:

$$H_0 = |H| + \frac{g_i}{V_0} \cdot \frac{K_1|H| + 2K_1K_2|H|^2}{1 + K_1|H| + K_1K_2|H|^2}$$

(S4) and thus

$$|H| = H_0 - \frac{g_i}{V_0} \cdot \frac{K_1|H| + 2K_1K_2|H|^2}{1 + K_1|H| + K_1K_2|H|^2}$$

(S5)

Let us now consider the optical rotation of each sample. Indeed, it must be the sum of the three independent contributions, due to the free host and the complexes respectively, i.e.:

$\vartheta_i = \Theta_H|H| + \Theta_{HG}|HG| + \Theta_{H2G}|H_2G| =$

$= \Theta_H(H_{0,i} - |HG| - 2|H_2G|) + \Theta_{HG}|HG| + \Theta_{H2G}|H_2G| =$

$= \Theta_H H_{0,i} + (\Theta_{HG} - \Theta_H)|HG| + (\Theta_{H2G} - 2\Theta_H)|H_2G|$

Now, if samples are prepared according to method A, by proper substitution we obtain:

$\vartheta_i = \Theta_H H_0/(1+ v_i/V_0) + (\Theta_{HG} - \Theta_H)\, G_0[(v_i/V_0)/(1 + v_i/V_0)]K_1|H|/(1 + K_1|H| + K_1K_2|H|^2) +$

$+ (\Theta_{H2G} - 2\Theta_H)\, G_0[(v_i/V_0)/(1 + v_i/V_0)]K_1K_2|H|^2/(1 + K_1|H| + K_1K_2|H|^2)$

which reduces to Equation 5 after trivial rearrangement, and considering that the product $\Theta_H H_0$ is the optical rotation ϑ_0 of the starting host solution. On the other hand, if samples are prepared according to method B, we obtain:

$\vartheta_i = \Theta_H H_0 + (\Theta_{HG} - \Theta_H)|HG| + (\Theta_{H2G} - 2\Theta_H)|H_2G| =$

$= \Theta_H H_0 + (\Theta_{HG} - \Theta_H)\, (g_i/V_0)K_1|H|/(1 + K_1|H| + K_1K_2|H|^2) +$

$+ (\Theta_{H2G} - 2\Theta_H)\, (g_i/V_0)K_1K_2|H|^2/(1 + K_1|H| + K_1K_2|H|^2)$

which, after trivial rearrangement, reduces to:

$$\vartheta_i = \vartheta_0 + \frac{g_i}{V_0} \cdot \frac{\Delta\Theta_{1:1}K_1|H| + \Delta\Theta_{2:1}K_1K_2|H|^2}{1 + K_1|H| + K_1K_2|H|^2}$$

(S6)

Thus, whenever samples are prepared by Method B, the iterative procedure for data fitting will provide the use of Equations S5 and S6.

Finally, when only the 2:1 complex is formed, all previous equations can be simplified as follows. If samples are prepared according to method A, then:

$$\vartheta_i = \frac{1}{1 + v_i/V_0}\left[\vartheta_0 + G_0(v_i/V_0)\frac{\Delta\Theta_{2:1}\beta_2|H|^2}{1 + \beta_2|H|^2}\right]$$

(S7) and

$$|H| = (1 + v_i/V_0)\left[H_0 - G_0(v_i/V_0)\frac{2\beta_2|H|^2}{1 + \beta_2|H|^2}\right]$$

(S8)

Differently, if samples are prepared according to method B, then:

$$\vartheta_i = \vartheta_0 + \frac{g_i}{V_0}\cdot\frac{\Delta\Theta_{2:1}\beta_2|H|^2}{1 + \beta_2|H|^2}$$

(S9) and

$$|H| = H_0 - \frac{g_i}{V_0}\cdot\frac{2\beta_2|H|^2}{1 + \beta_2|H|^2}$$

(S10)

On the conditions for the exclusive detection of the 2:1 complex

Whenever the 1:1 complex is so much less stable than the 2:1 complex, that it cannot be detected in a significant amount, the following algebraic condition must be verified:

$K_1|H| << 1 + K_1K_2|H|^2$

We may assume that this condition is reasonably fulfilled if the concentration of the 1:1 complex is always lower than 5% of the concentration of the 2:1 complex, i.e.:

$|HG| \leq 0.05|H_2G|$ or $|H_2G| \geq 20|HG|$

and because

$K_2 = |H_2G|/(|H||HG|)$

one easily obtains:

$K_2 \geq 20/|H|$

In our experiments, $|H|$ can never be larger than 0.0025 M (which is the analytical concentration of the mother solution of the free host); consequently, it must be $K_2 \geq 8000$ M^{-1}.

Synthesis of preCA

The synthesis was accomplished using a modification of the procedure reported by Tunstad et al. [S3]. Equimolar amounts of resorcinol (3.3 g, 30 mmol) and butyraldehyde (2.7 mL, 2.16 g, 30 mmol) were dissolved in ethanol (24 mL), then 32% conc. HCl (6 mL) was slowly added dropwise,

and the solution was allowed to reflux at 93° overnight under inert atmosphere (Ar). The reddish solution obtained was cooled to rt and poured into water (250 mL). The precipitated crude product was collected by filtration and purified by column chromatography (silica gel, dichloromethane/methanol 92:8 v/v). Yield 60 % (2.96 g). IR (nujol): ῦ (cm^{-1}) 3534, 3476, 3304, 2727, 1614, 1508. ^{1}H-NMR (DMSO-d_6): δ (ppm) 1.00 (t, 12 H, J = 7.2 Hz, -CH_3), 1.34 (m, 8 H, -<u>CH_2</u>CH_3), 2.21 (m, 8 H, -<u>CH_2</u>CH<) 4.35 (t, 4 H, J = 7.2 Hz, -CH<), 6.13 (s, 4 H, ArH), 7.23, 7.27 (2s, 2H + 2H, ArH), 9.38, 9.65 (2s, 4H + 4H, ArOH). It is worth noting the splitting of the signal relevant to both the phenolic groups and to the aromatic H at the lower rim, which can be attributed to the occurrence of a pinched conformation with a C2v symmetry, stable enough to interconvert only very slowly with the perfect cone conformation (C4v symmetry). Elem. Anal.: C 73.12 %, H 7.40 % (calcd. for $C_{40}H_{48}O_8$: C 73.15 %, H 7.37 %). Because the reaction is lead under thermodynamic control conditions, according to Högberg's studies [S3,S4], the product assumes a "*cis-cis-cis*" stereochemistry (Figure S1).

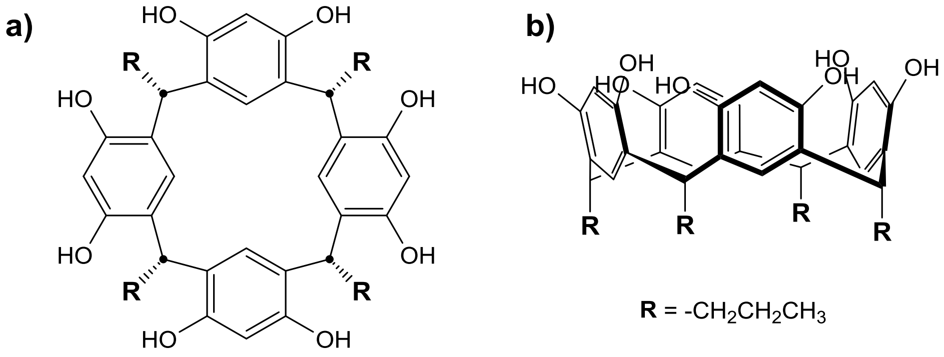

Figure S1: "*cis-cis-cis*" structure of preCA: a) top view; b) side view.

Synthesis of CAP

The synthetic procedure was adapted from refs [S5-S7] and [S8]. In detail, L-proline (2.32 g, 20 mmol) and preCA (2.62 g, 3 mmol) were dissolved in methanol (48 mL); then 37% conc. formaldehyde was added (2.1 mL, 75 mmol), and the mixture was kept at 40 °C for 3 days under inert atmosphere (Ar) and magnetic stirring. The solid product formed is collected by vacuum filtration, washed with ice-cold diethyl ether, and used as such without further purification. Yield 87% (3.04 g). IR (nujol): ῦ (cm^{-1}) 3196, 2727, 2674, 2607, 1727, 1613. ^{1}H-NMR (DMSO-d_6): δ (ppm) 0.94 (t, 12 H, J = 7.2 Hz, -CH_3), 1.24 (m , 8 H, -<u>CH_2</u>CH_3), 1.60-1.90 (m, 12 H, H(3,4,4')Pro), 2.10-2.35 (m, 12 H, H(3')Pro overlapped with -<u>CH_2</u>CH<), 2.63 (m, 4 H, H(5)Pro), 3.05 (m, 4 H, H(5')Pro), 3.58

(m, 4H, H(2)Pro), 3.96, 4.03 (2d, 4 H + 4 H, *J* = 14.2 Hz, Ar-CH_2-Pro), 4.24 (t, 4 H, *J* = 7.2 Hz, -CH<), 7.49 (s, 4 H, ArH). The correct attribution of the signals has been confirmed by COSY. Elem. Anal.: C 65.94 %, H 7.31 %, N 4.80 % (calcd. for $C_{64}H_{84}N_4O_{16}$: C 65.96 %, H 7.27 %, N 4.81 %).

The ^{1}H-NMR spectrum of CAP^{-1} recorded in D_2O shows a dramatic loss of resolution of all the signals and a general upfield shift: δ (ppm) 0.82 (br s, 12 H, -CH_3), 1.14 (br s , 8 H, -$\underline{CH_2}CH_3$), 1.54, 1.73, 1.91, 2.02, 2.23, 2.63, 2.79 (br s, 36 H, H(2,3,4,5)Pro overlapped with-$\underline{CH_2}$CH<), 3.81, 4.06 (br s, 4 H + 4 H, Ar-CH_2-Pro), 4.21 (br s, 4 H, -CH<), 7.01 (s, 4 H, ArH).

The ^{1}H-NMR spectrum of the 8·CAP^{-1} complex recorded in D_2O shows the following signals: δ (ppm) 0.71 (br s, 12 H, -CH_3), 1.03 (br s , 8 H, -$\underline{CH_2}CH_3$), 1.54, 1.73, 1.92, 2.03, 2.20, 2.64 (br s, 42 H, H(2,3,4,5)Pro overlapped with-$\underline{CH_2}$CH< and aliphatic H of the guest), 2.91, 3.03 (br s , 4 H, H(2)Pro) 3.77, 4.04 (br s, 4 H + 4 H, Ar-CH_2-Pro), 4.15 (br s, 4 H, -CH<), 6.98 (s, 4 H, ArH).

References

S1 Lo Meo, P.; D'Anna, F.; Riela, S.; Gruttadauria, M.; Noto, R. *Tetrahedron Lett.* **2006,** *47,* 9099–9102. doi:10.1016/j.tetlet.2006.10.078

S2 Lo Meo, P.; D'Anna, F.; Riela, S.; Gruttadauria, M.; Noto, R. *Tetrahedron* **2007,** *63,* 9163–9171. doi:10.1016/j.tet.2007.06.065

S3 Tunstad, L. M.; Tucker, J. A.; Dalcanale, E.; Weiser, J.; Bryant, J. A.; Sherman, J. C.; Helgeson, R. C.; Knobler, C. B.; Cram, D. J. *J. Org. Chem.* **1989,** *54,* 1305–1312. doi:10.1021/jo00267a015

S4 Lide, D. R., Ed. *Handbook of Chemistry and Physics,* 86 ed.; CRC Press: Boca Raton, FL, 2005.

S5 Hagan, K. A.; O'Farrell, C. M.; Wenzel, T. J. *Eur. J. Org. Chem.* **2009,** 4825–4832. doi:10.1002/ejoc.200900674

S6 O'Farrell, C. M.; Hagan, K. A.; Wenzel, T. J. *Chirality* **2009,** *21,* 911–921. doi:10.1002/chir.20688

S7 O'Farrell, C. M.; Wenzel, T. J. *Tetrahedron: Asymmetry* **2008,** *19,* 1790–1796. doi:10.1016/j.tetasy.2008.07.023

S8 Shahgaldian, P.; Pieles, U.; Hegner, M. *Langmuir* **2005,** *21,* 6503–6507. doi:10.1021/la0503101
